# Palmitate induces endoplasmic reticulum stress and autophagy in mature adipocytes: Implications for apoptosis and inflammation

**DOI:** 10.3892/ijmm.2015.2085

**Published:** 2015-01-30

**Authors:** JIAJING YIN, YUFAN WANG, LIPING GU, NENGGUANG FAN, YUHANG MA, YONGDE PENG

**Affiliations:** Department of Endocrinology, Shanghai First People’s Hospital, Shanghai Jiao Tong University, Shanghai 200080, P.R. China

**Keywords:** palmitate, autophagy, endoplasmic reticulum stress, inflammation, adipocytes

## Abstract

Endoplasmic reticulum (ER) stress and inflammation induced by obesity lead to adipocyte dysfunction, with the impairment of the insulin pathway. Recent studies have indicated that understanding the physiological role of autophagy is of great significance. In the present study, an *in vitro* model was used in which 3T3-L1 adipocytes were pre-loaded with palmitate (PA) to generate artificially hypertrophied mature adipocytes. PA induced an autophagic flux, determined by an increased microtubule-associated protein 1 light chain 3 (LC3)-II formation, as shown by western blot analysis and fluorescence microscopy, and was confirmed using transmission electron microscopy (TEM). Using TEM and western blot analysis, we observed increased ER stress in response to PA, as indicated by the increased levels of the ER stress markers, BiP, activating transcription factor 4 (ATF4) and C/EBP homologous protein (CHOP), and the phosphoralytion of eukaryotic translation initiation factor 2α and c-Jun N-terminal kinase (JNK). Of note, we observed that the PA-induced ER stress occurred prior to the activation of autophagy. We confirmed that autophagy was induced in response to JNK-dependent ER stress, as autophagy was suppressed by treatment with the ER stress inhibitor, 4-phenyl butyrate (4-PBA), and the JNK inhibitor, SP600125. Upon the inhibition of autophagy using chloroquine (CQ), we observed exacerbated ER stress and an increased level of cell death. Importantly, to determine whether autophagy is linked to inflammation, the autophagy inhibitor, 3-methyladenine (3-MA) was used. The inhibition of autophagy led to a further increase in the PA-induced expression of monocyte chemoattractant protein-1 (MCP-1) and interleukin-6 (IL-6). Consistently, such an increase was also observed following treatment with SP600125. In conclusion, our data indicate that PA elicits a ER stress-JNK-autophagy axis, and that this confers a pro-survival effect against PA-induced cell death and stress in hypertrophied adipocytes. The JNK-dependent activation of autophagy diminishes PA-induced inflammation. Therefore, the stimulation of autophagy may become a method with which to attenuate adipocyte dysfunction and inflammation.

## Introduction

Obesity results in a low-grade and chronic inflammatory state and β cells, adipocytes and other metabolic cells respond to the excess inflow of nutrients and energy by stress signals that trigger inflammation (1,2). Adipocytes are not only storage units for triglycerides, but also influence systemic lipid homeostasis through the production and release of adipocyte-specific and adipocyte-enriched hormonal factors, inflammatory mediators, such as the pro-inflammatory cytokines, tumor necrosis factor-α (TNF-α), interleukin (IL)-6 and monocyte chemoattractant protein-1 (MCP-1), and anti-inflammatory adiponectin (3). The dysregulation of lipolysis by the increased expression of adipose inflammatory cytokines is an important factor contributing to systemic insulin resistance (4). The pathophysiology of adipocyte dysfunction under stress is complex, therefore suggesting that the adaptive mechanisms that operate in stressed adipocytes may have important implications for understanding the mechanisms of obesity and other metabolic disorders.

Autophagy is an evolutionarily conserved lysosome-dependent system in found eukaryotes that regulates the turnover of cellular proteins and organelles. During autophagy, target proteins or organelles are delivered into double-membrane autophagosomes for lysosomal degradation (5). This self-eating system has been shown to control a variety of functions, including the control of innate and adaptive immune responses by regulating cytokine production (6) and to combat persistent endoplasmic reticulum (ER) stress (7). Deficient autophagy by the suppression of autophagy-related gene 7 (ATG7) renders hepatocytes vulnerable to ER stress and insulin resistance, and conversely, the restoration of hepatic autophagy by means of ATG7 overexpression improves insulin sensitivity (8). Macrophages derived from ATG16L1-deficient mice have been shown to produce higher levels of IL-1β (9), and mice with a conditional deletion of ATG7 in the intestinal epithelium show an enhanced IL-1β expression (10). Changes in adipose autophagy in obesity and metabolic disorders are now rapidly being characterized; however, the precise role of autophagy remains unclear and may depend on the disease state and experimental model under investigation. For example, Ost *et al* (11) demonstrated that the autophagosome content was increased in isolated adipocytes derived from obese and diabetic patients. By contrast, in rodents, autophagy has been shown to be reduced both *in vitro* and in the adipose tissue of animals (12).

ER stress and pro-inflammatory cytokines both play a relevant role in adipose tissue inflammation (10,13). However, our understanding of the molecular mechanisms of autophagy during inflammation and stress remains incomplete. In this study, we used palmitate (PA) to generate artificially hypertrophied mature adipocytes and examined whether there was a resulting change in autophagy and inflammation. Importantly, we used methodological approaches to examine the changes in autophagy. We also examined the crosstalk between autophagy and ER stress or inflammation. We aimed to determine whether autophagy plays a protective or detrimental role in stressed adipocyte inflammation in obesity.

## Materials and methods

### Reagents and antibodies

Fetal bovine serum (FBS), Dulbecco’s modified Eagle’s medium (DMEM), 1% (v/v) streptomycin/penicillin and TRIzol reagent were from Gibco Invitrogen, (Grand Island, NY, USA); the Superscript III First-strand Synthesis System was from Promega (Madison, WI, USA). Rapamycin (RAP) was purchased from Merck Bioscience (Darmstadt, Germany). PA (P0500), bovine serum albumin (BSA) (albumin, endotoxin), chloroquine (CQ), 4-phenyl butyrate (4-PBA), 3-methyladenine (3-MA), SP600125 and 3-(4,5-dimethylthiazol-2-yl)-2,5-diphenyltetrazolium bromide (MTT) were from Sigma-Aldrich (St. Louis, MO, USA). The PageRuler™ Prestained Protein Ladder was from Thermo Scientific (Kalamazoo, MI, USA). Complete protease inhibitor mixture and immunoblot polyvinylidene difluoride (PVDF membranes were from Roche Diagnostics (Barcelona, Spain). RIPA lysis buffer and the BCA protein assay kit were from Beijing ComWin Biotech Co., Ltd. (Beijing, China). Western Chemiluminescent HRP substrate was purchased from Millipore (Billerica, MA, USA). Rabbit anti-microtubule-associated protein 1 light chain 3 (LC3) 1:1,000 (no. 2775), rabbit anti-activating transcription factor 4 (ATF4) 1:1,000 (no. 11815), rabbit anti-C/EBP homologous protein (CHOP) 1:1,000 (no. 5554), rabbit anti-p-eukaryotic translation initiation factor 2α (eIF2α) 1:1,000 (no. 3398), rabbit anti-p-c-Jun N-terminal kinase (JNK) and total JNK 1:1,000 (no. 9912) were from Cell Signaling Technology (Beverly, MA, USA). Goat polyclonal anti-BiP 1:200 (sc-1050) and goat polyclonal anti-glyceraldehyde 3-phosphate dehydrogenase (GAPDH) 1:200 (sc-48166) were from Santa Cruz Biotechnology, Inc. (Santa Cruz, CA, USA).

### Cell culture and PA treatment

The 3T3-L1 cells were obtained from the American Type Culture Collection (ATCC; Rockville, MD, USA). The cells were seeded and fed every 2 days in DMEM containing 25 mM glucose supplemented with 50 U/ml penicillin, 50 *μ*g/ml streptomycin, 100 mM MEM sodium pyruvate and 10% FBS. The cells were grown under 5% CO_2_ at 37°C. At confluence, differentiation was induced by the addition of medium containing 0.5 mM isobutylmethylxanthine, 1 *μ*M dexamethasone (Sigma-Aldrich) and 10 *μ*g/ml insulin. After 48 h, this mixture was replaced with fresh medium, and this was changed every 2 days. On days 6–8 after the induction of adipocyte differentiation, the cells were used for the experiments. PA/BSA conjugates were prepared as previously described (14). PA was dissolved at 70°C in 0.1 M NaOH to obtain a 100 mM stock solution. A 5% (w/v) solution of free fatty acid (FFA)-free BSA was prepared in double distilled water. Subsequently, a 5 or 10 mM PA/BSA mixture was prepared by suitable combination of the 2 above-mentioned solutions. Finally, the mixture was further diluted in serum-free medium to obtain the required final concentrations of 0.5 mM PA/0.5% BSA or 1.0 mM PA/0.5% BSA.

### Transmission electron microscopy (TEM)

After the indicated treatments, mature 3T3-L1 adipocytes were fixed in phosphate buffer (pH 7.4) containing 2.5% glutaraldehyde and 2% paraformaldehyde at room temperature for 60 min. The cells were post-fixed in 1% OsO_4_ at room temperature for 60 min, dehydrated through graded ethanol solutions, and embedded in Quetol 812 (Nisshin EM Co., Tokyo, Japan). Areas containing cells were block-mounted and cut into 70 nm sections that were stained with uranyl acetate (saturated aqueous solution) and lead citrate and examined under a transmission electron microscope (H-7100; Hitachi, Ibaraki, Japan).

### Immunofluorescence

The cells were grown on round glass coverslips in 35 mm cell culture dishes. Following a 20-min fixation with pre-chilled methanol, the coverslips were washed with phosphate-buffered saline (PBS) and permeabilized with 0.2% Triton X-100-PBS for 30 min. The coverlips were then incubated with rabbit polycolonal LC3 primary antibodies (1:200) at 37°C for 90 min in the dark, followed by 3 washes (10 min each) in PBS. Subsequently, the coverlips were incubated with goat-anti rabbit IgG-FITC secondary antibodies (1:1,000) at 37°C for 1 h in dark and washed 3 times (10 min each time) in PBS. Digital images were obtained at an original magnification of x20 using a Nikon C1 confocal microscope, with NIS-Element software (Nikon, Melville, NY, USA).

### Western blot analysis

The cells were washed with PBS and lysed in lysis buffer (0.5% Triton X-100, 10 mM HEPES pH 7.9, 50 mM NaCl, 100 mM EDTA, 0.5 M sucrose) containing 0.1% protease inhibitor cocktail (Roche Diagnostics). The lysates were then incubated on ice for 30 min and centrifuged at 8,000 × g for 10 min. Equal amounts of protein were subjected to sodium dodecyl sulfate-polyacrylamide gel electrophoresis (SDS-PAGE; 10–15%), transferred onto PVDF membranes. Molecular weights were estimated by comparison with a pre-stained protein ladder. Non-specific binding was blocked using 5% skim milk. The membranes were then incubated with specific primary antibodies overnight at 4°C. The membranes were washed with PBS-Tween-20 and incubated with peroxidase-conjugated secondary antibodies [anti-rabbit IgG, HRP-linked sntibody 1:5,000 (no. 7074); Cell Signaling Technology; and mouse anti-goat IgG, HRP-linked antibody 1:5,000 (no. sc-2354); Santa Cruz Biotechnology, Inc.] Protein bands were detected using the Western Chemiluminescent HRP substrate (Millipore). Immunoblots were quantified by densitometric analysis using ImageTool 3.0 software. The quantification of protein phosphorylation was normalized to the corresponding total protein expression, and the relative expression level of a certain protein was normalized to GAPDH.

### Reverse transcription-quantitative (real-time) polymerase chain reaction (RT-qPCR)

Total RNA was extracted using TRIzol reagent (Invitrogen); samples of 1 *μ*g total RNA were reverse transcribed into cDNA using a cDNA synthesis kit (Promega). Quantitative PCR (qPCR) was performed using an ABI 7900 sequencer (Life Technologies, Carlsbad, CA, USA) with the SYBR-Green PCR Master Mix (Promega) and d(N)_6_ random hexamer with primers obtained from Invitrogen. The thermocycling parameters were as follows: 95°C for 10 min, 40 cycles of 95°C for 15 sec, 60°C for 1 min. Each sample was run in triplicate and normalized against *36B4* RNA. The fold changes were determined using the ΔΔCt method. The primers (mouse) used were as follows: IL-6 forward, 5′-CTGGGAAA TCGTGGAAATG-3′ and reverse, 5′-CCAGAGGAAATTTT CAATAGGC-3′; MCP-1 forward, 5′-AGCCAACTCTCACTG AAGCCA-3′ and reverse, 5′-AGTAGCAGCAGGTGAGT GGG-3′; and 36B4 forward, 5′-CGACCTGGAAGTCCAA CTAC-3′ and reverse, 5′-ATCTGCTGCATCTGCTTG-3′.

### Cell viability

Cell viability was assessed by MTT assay. Briefly, mature adipocytes were seeded in a 96-well plate and incubated under different conditions. The culture medium was then removed, and 200 *μ*l/well were diluted in MTT solution (0.5 mg/ml) in PBS for 1 h at 37°C. During the incubation time, MTT was converted to an insoluble formazan. At the end of the incubation time, the medium containing MTT was aspirated, and each well was gently washed with PBS. Dimethyl sulfoxide (DMSO) was then used to solubilize the precipitated formazan, and the concentration was determined using a microplate reader at OD 570 nm.

### Statistical analysis

The results are expressed as the means ± SEM. Comparisons of a single variable in >2 groups were analyzed by one-way ANOVA followed by Tukey’s multiple comparison tests (GraphPad Prism). Statistical analysis was performed using the paired and unpaired t-test between 2 groups, using SPSS 12.0 software (SPSS, Inc., Chicago, IL, USA). Values of P<0.05 were considered to indicate statistically significant differences.

## Results

### Autophagy is activated in response to PA

**Figure 1:**
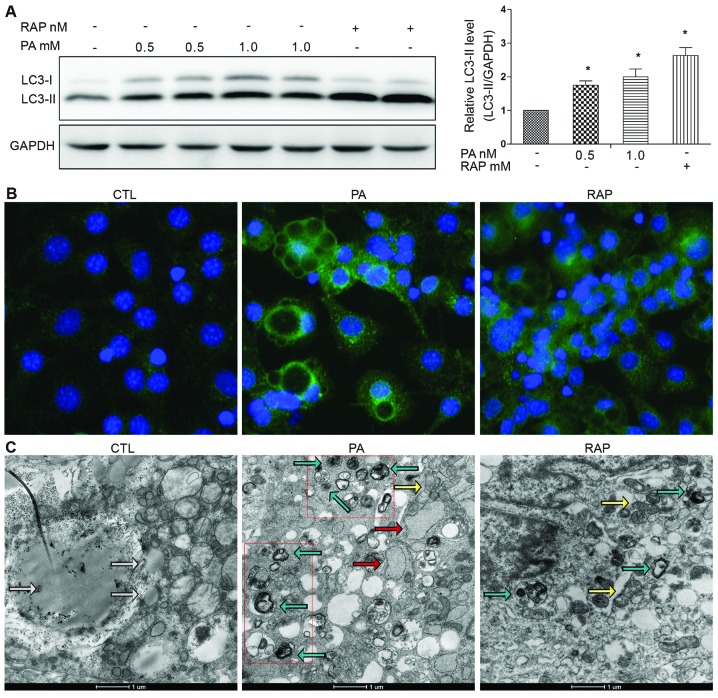
Autophagy is activated in response to treatment with palmitate (PA). (A) Adipocytes were treated with or without PA (0.5 and 1.0 mM) or rapamycin (RAP; 100 nM) for 12 h, followed by the analysis of endogenous LC3 by western blotting. The proteins were quantified and normalized to GAPDH (right panel). Results are the means ± SEM of 3 to 5 separate experiments. ^*^P<0.05 vs. control group (0.5% BSA). (B) Indirect immunofluorescence of LC3 was performed on mature adipocytes exposed to 0.5 mM PA or 100 nM RAP for 12 h; the green signal represents the LC3 levels (magnification, x20). Control cells (CTL) were treated with 0.5% BSA for 12 h. (C) Electron microscopy of mature adipocytes exposed to 0.5 mM PA or 100 nM RAP for 12 h. Control cells (CTL) were treated with 0.5% BSA for 12 h. Scale bars represent 1 *μ*m. White arrow represent lipid droplets; red rectangle and blue arrows represent autophagosomes and autolysosomes of double-membrane, single-membrane and multivesicular body-like vesicles; red arrows represent a dilated endoplasmic reticulum; yellow arrows represent the mitochondria.

As the lipidation of LC3 and its association with autophagsome membranes has been established as a useful marker of autophagy (15), we detected LC3 expression by western blot analysis and fluorescence microscopy. A reliable marker of autophagy is the conversion of the ATG protein, LC3, from a soluble form (LC3-I) to a lipidized form (LC3-II), which stably associates with the membranes of autophagosomes (16). This conversion can be detected by measuring the accumulation of the LC3-II form. Our results demonstrated that treatment with PA increased LC3-II formation in response to treatment with PA (0.5 or 1.0 mM) for 12 h (Fig. 1A). RAP, a known mammalian target of rapamycin (mTOR) pathway inhibitor, is known to induce autophagy (17). RAP was used as a positive control. We observed significant LC3-II accumulation following treatment with RAP (100 nM) for 12 h (Fig. 1A). The number of LC3-II puncta was increased and the LC3-II puncta were clearly visible in the PA (0.5 mM)-treated group compared with the control group (cells treated with 0.5% BSA), shown as green fluorescent granules in the cytoplasm, mainly around the nucleus. The number of LC3-II puncta was also increased in the cells treated with RAP (Fig. 1B).

To gain insight into the morphological changes induced by PA, electron microscopy was performed using the mature 3T3-L1 adipocytes. Treatment with PA (0.5 mM) for 12 h induced the formation of autophagosomes, which were recognized at the ultrastructural level as double-membrane vacuolar structures containing visible cytoplasmic contents. Autolysosomes were recognized as single-membrane vacuolar structures containing high-density materials and some contained multivesicular body-like vesicles (Fig. 1C). These characteristics were also observed in the cells treated with RAP (100 nM) for 12 h. Abundant lipid droplets, but rare evidence of autophagosomes were observed in the control cells (Fig. 1C).

### PA induces ER stress and subsequent autophagy

**Figure 2:**
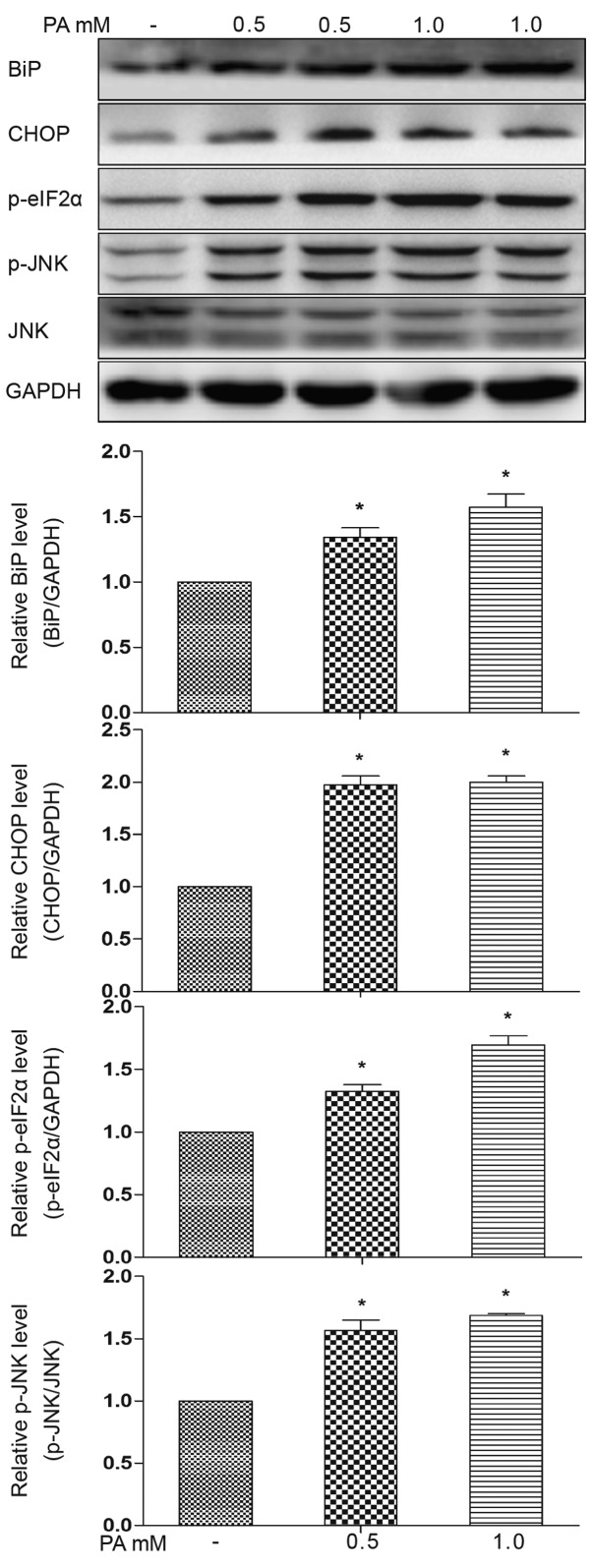
Palmitate induces endoplasmic reticulum (ER) stress in mature adipocytes. (A) Mature 3T3-L1 adipocytes were treated with or without palmitate (0.5 or 1.0 mM) for 12 h, and western blot analysis was performed with the indicated antibodies to BiP, C/EBP homologous protein (CHOP), phosphoralyted eukaryotic translation initiation factor 2α (eIF2α) and c-Jun N-terminal kinase (JNK). Proteins were quantified and normalized to GAPDH. Representative blots and quantifications are shown. Results are the means ± SEM of 3 to 5 separate experiments. ^*^P<0.05 vs. control group (0.5% BSA).

ER stress is a potential molecular mechanism of lipotoxicity, and we thus examined whether PA induces ER stress in mature adipocytes. We observed an increase in ER stress protein markers, evidenced by an increase in the expression of BiP, CHOP and eIF2α phosphoralytion, as well as in JNK phosphoralytion following treatment with PA (0.5 or 1.0 mM) for 12 h (Fig. 2). These results suggest the activation of the PKR-like endoplasmic reticulum kinase (PERK)-associated unfolded protein response (UPR) pathways, as well as inositol-requiring enzyme 1 (IRE1) pathways. Increased CHOP expression occurs downstream of the main pathways activated following ER stress, namely PERK, ATF6 and IRE1 (18). In addition, electron microscopy was performed on the PA-treated adipocytes (for 12 h) and revealed a dilated ER (Fig. 1C, red arrow).

**Figure 3:**
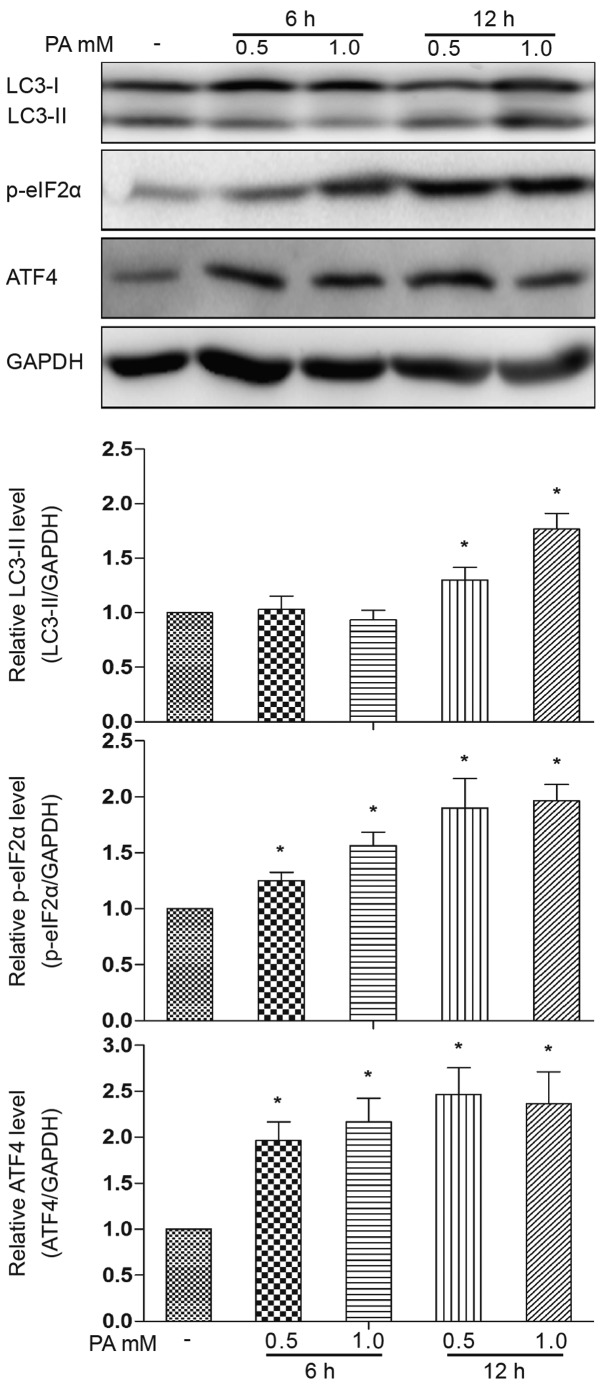
Palmitate (PA) induces autophagy subsequent to the activation of endoplasmic reticulum (ER) stress. Mature 3T3-L1 adipocytes were treated with 0.5 or 1.0 mM PA for 6 and 12 h. Western blot analysis was performed using antibodies to LC3, phosphoralyted eukaryotic translation initiation factor 2α (eIF2α) and activating transcription factor 4 (ATF4). Proteins were quantified and normalized to GAPDH. Representative blots and quantifications are shown. Results are the means ± SEM of 3 to 5 separate experiments. ^*^P<0.05 vs. control group (0.5% BSA for 12 h).

**Figure 4:**
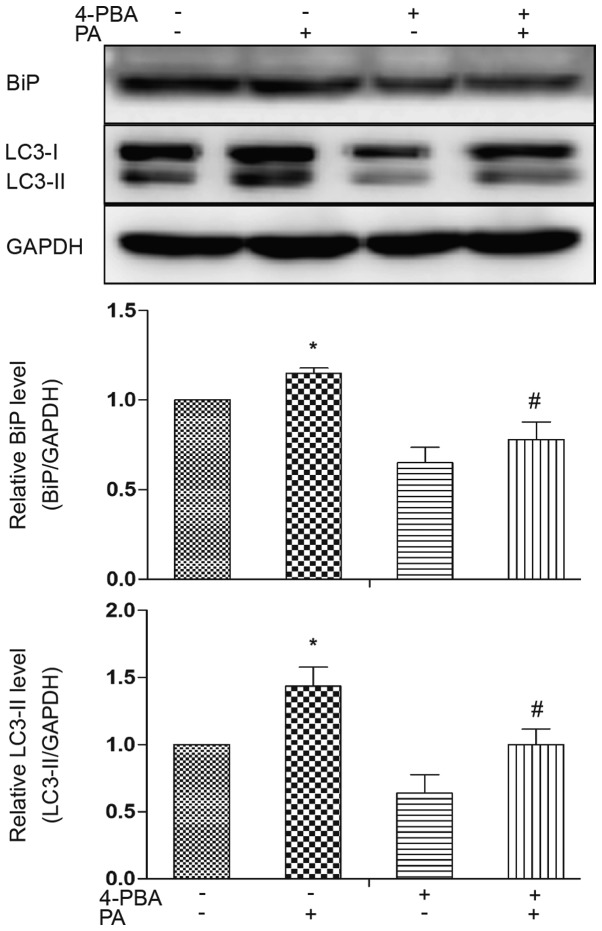
Palmitate (PA) induces autophagy through the the endoplasmic reticulum (ER) stress-dependent pathway. Mature 3T3-L1 adipocytes were pre-treated with or without 4-phenyl butyrate (4-PBA; 7.5 mM) for 1 h, followed by treatment with PA (0.5 mM) for 12 h. Western blot analysis was performed with antibodies to BiP and LC3. Proteins were quantified and normalized to GAPDH. Representative blots and quantifications are shown. Results are the means ± SEM of 3 to 5 separate experiments. ^*^P<0.05 vs. control group (0.5% BSA); ^#^P<0.05 vs. PA-treated group.

We further addressed the cause-effect relationship among ER stress and autophagy. The phosphorylation of eIF2α and the ATF4 protein expression level were increased following treatment with PA (0.5 and 1.0 mM) for 6 h (Fig. 3). No obvious accumulation of LC3-II was observed following treatment with PA for 6 h, but only at 12 h of treatment. Based on this observation, we then investigated the association between ER stress and autophagy by treating the adipocytes with 4-PBA, an ER stress inhibitor. ER stress was ameliorated by treatment with 4-PBA, as evidenced by a decrease in the expression level of the ER stress marker, BiP (Fig. 4). Western blot analysis revealed that LC3-II accumulation was decreased in the presence of 4-PBA compared to treatment with PA alone (Fig. 4). These results indicate a potential causal involvement of ER stress in the induction of autophagy induced by PA.

### The inhibition of autophagy renders cells susceptible to ER stress and apoptosis

**Figure 5:**
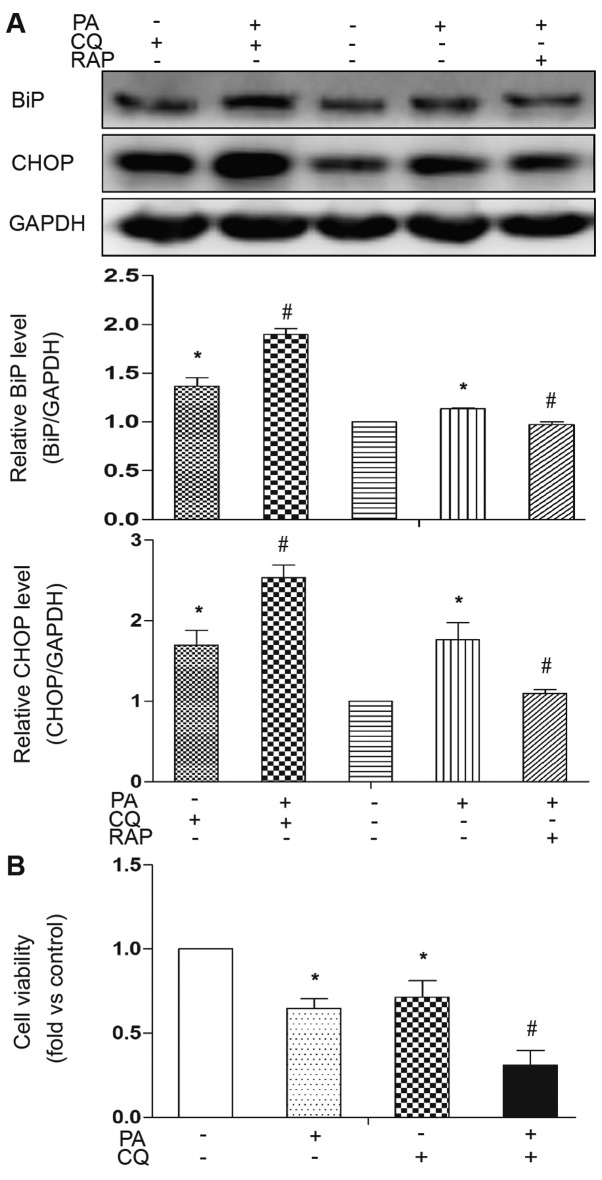
The inhibition of autophagy renders cells susceptible to endoplasmic reticulum (ER) stress and apoptosis. (A) Mature 3T3-L1 adipocytes were pre-treated with or without rapamycin (RAP) (100 nM) and/or chloroquine (CQ) (10 *μ*M) for 1 h, followed by treatment with palmitate (PA) (0.5 mM) for 12 h. Western blot analysis and quantification of protein expression was performed with the indicated antibodies. Data are presented as the means ± SEM (n=3). ^*^P<0.05 vs. control group (0.5% BSA); ^#^P<0.05 vs. PA-treated group. (B) Cell viability of mature 3T3-L1 adipocytes pre-treated with or without CQ (10 *μ*M) for 1 h, followed by treatment with PA (0.5 mM) for 24 h by MTT assay. Bars represent the means ± SD, n=6; ^*^P<0.05 vs. control group (0.5% BSA); ^#^P<0.05 vs. PA-treated group.

To address the role of PA-induced autophagy in mature adipocytes, the cells were co-treated with CQ, a lysosome inhibitor, to block the autophagic flux, as previously described (19). The pharmacological inhibition of autophagy by CQ significantly increased the expression levels of the PA-induced ER stress markers, BiP and CHOP, indicating the extent of cellular stress when PA-induced autophagy was inhibited (Fig. 5A). Conversely, treatment with RAP to induce autophagy reversed these effects in response to PA (Fig. 5A). In addition, the expression levels of the ER stress markers were also increased by treatment with CQ without PA treatment, indicating that basal autophagy plays a role against cellular stress (Fig. 5A). The detrimental effects of the inhibition of PA-induced autophagy were confirmed using a cell viability assay. Cell viability significantly decreased by co-treatment with PA and CQ (Fig. 5B). Treatment with CQ alone also decreased cell viability, indicating that basal autophagy plays a role against cell death. These results demonstrate that the activation of autophagy is an adaptive response to PA and functions as a mechanism for cellular self-protection against ER stress and cell death.

### ER stress-induced autophagy is partially JNK-dependent and mediates the limitation of PA-induced inflammatory cytokine expression

**Figure 6:**
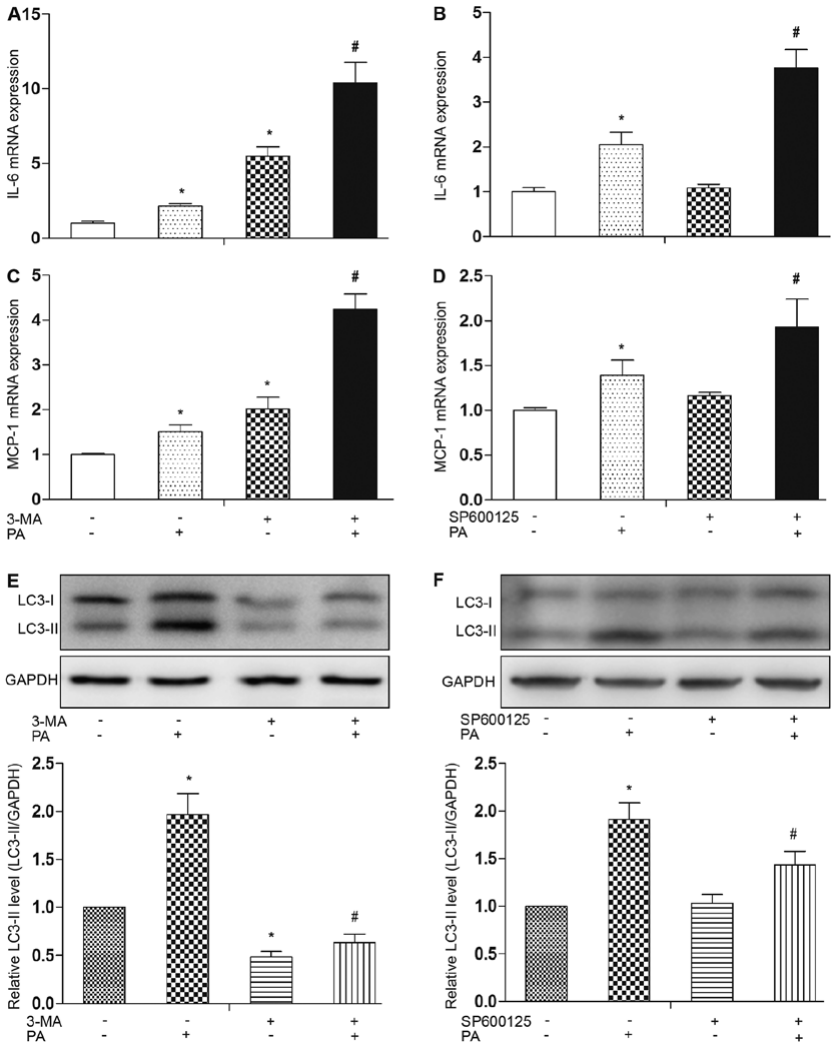
Autophagy limits palmitate (PA)-induced inflammatory cytokine expression. Mature 3T3-L1 adipocytes were pre-treated with or without 3-methyladenine (3-MA) (10 mM) or SP600125 [c-Jun N-terminal kinase (JNK) inhibitor, 10 *μ*M] for 1 h, followed by treatment with PA (0.5 mM) for 12 h. (A-D) mRNA expression levels of monocyte chemoattractant protein-1 (MCP-1) and interleukin-6 (IL-6) were analyzed by qPCR. (E and F) Western blot analysis and quantification of protein expression was performed using LC3 antibodies. Data are presented as the means ± SEM of 3 to 5 separate experiments. ^*^P<0.05 vs. control group (0.5% BSA); ^#^P<0.05 vs. PA-treated group.

Pre-loading of the cells with 0.5 mM/l of the saturated fatty acid, PA, for 12 h resulted in an increase in the mRNA expression levels of the inflammatory cytokines, IL-6 and MCP-1, in the mature adipocytes (Fig. 6A and C). In order to determine the role of autophagy in PA-induced inflammatory cytokine expression, we pre-treated the adipocytes with the autophagy inhibitor, 3-MA, for 1 h followed by treatment with PA for 12 h. 3-MA effectively inhibited the induction of autophagy by PA, as evidenced by the decrease in LC3-II accumulation (Fig. 6E). RT-qPCR revealed that treatment with 3-MA further increased the mRNA expression levels of MCP-1 and IL-6 compared to treatment with PA alone (Fig. 6A and C). Since the induction of autophagy by ER stress is in part mediated by JNK activation (20), we then examined the role of JNK in PA-induced autophagy in mature adipocytes using the specific JNK inhibitor, SP600125. As anticipated, the enhanced accumulation of LC3-II was decreased by pre-treatment with SP600125 compared to treatment with PA alone (Fig. 6F). Importantly, the expression of IL-6 and MCP-1 was further increased by SP600125 in the PA-treated adipocytes (Fig. 6B and D). These results demonstrate that PA-induced autophagy is partially JNK-dependent and that the activation of JNK-dependent autophagy plays a role in limiting inflammatory cytokine expression.

## Discussion

It has become well accepted that changes in inflammation in adipocytes and the infiltration of adipose tissue by immune cells are key characteristics of obesity in animal models and humans (21). Increased exposure to fatty acids, whether due to the increased fat content of modern diets or aberrant lipolysis in adipocytes has been suggested as one of the key activators of both altered metabolic and immune signaling in obesity. Indeed, the mechanisms through which fatty acids contribute to the emergence of inflammation and the adaptive response remain an important avenue for further research. In this study, we used PA treatment as an *in vitro* method to generate artificially hypertrophied adipocytes and to examine the effects on the cells, including autophagy, ER stress and inflammation, as well as the crosstalk between these cellular events. We demonstrate that autophagy plays a role in limiting cellular stress and inflammation as a positive response to PA in adipocytes.

Autophagy is known to play a vital role in adipose tissue to maintain the cellular concentration of ATP in diabetic adipocytes (11). Accordingly, it has been demonstrated that autophagy is essential for the completion of adipogenesis, functioning as a potential survival mechanism for cells during the differentiation process (22). On the other hand, the ER stress-induced downregulation of adiponectin appears to be mediated by an autophagy-dependent mechanism (23). Indeed, multiple characteristics of autophagy, such as an increase the number of autophagic vacuoles (AVs), has been observed in human obesity and type 2 diabetes (22). Our data demonstrated that autophagy was activated in mature adipocytes in response to PA. We used temporal analysis of LC3-II formation to show a rapid and transient effect of PA on autophagosome formation in adipocytes. Our data from TEM and LC3 staining clearly indicated that PA induced an autophagic flux in these cells. Autophagy can be activated under various circumstances, as occurs with the Toll-like receptor (TLR) (24). Both adipocytes and adipose tissue inflammatory cells express TRL4 (25), and FFA has been suggested to be a trigger of metabolism-associated inflammation through TLR2/4 (26). We hypothesized that the effects of faty acids on autophagy may differ substantially depending on the fatty acid used, the cell type examined and the duration of treatment. For example, as previously demonstrated, 8 h treatment of PA was sufficient to activate autophagosome formation in human hepatic cells, whereas prolonged treatment beyond 24 h blocked the autophagic flux and increased ER stress (27). In this study, we demonstrate that PA induces ER stress in mature adipocytes and an increase in the autophagic flux, which occurs at least in part in response to ER stress and is JNK-dependent. This conclusion was based on the use of the molecular chaperone, 4-PBA, which abolishes UPR induction, as well as on the use of a JNK inhibitor (SP600125). Indeed, it has been demonstrated that in other cell types in response to various stimuli, ER stress initiates the UPR which leads to a myriad of compensatory cellular effects, one of which is the induction of autophagy (28). Thus, our data confirm that in mature adipocytes, PA elicits this ER stress-JNK-autophagy axis, and based on previously reported data (29), it was important to determine the functional consequences of the PA-induced increase in the autophagic flux.

It has been postulated that ER stress-induced autophagy may have evolved as a mechanism used by cells to dispose of misfolded proteins that cannot be degraded by endoplamic reticulum-related degradation, consequently, assisting ER homeostasis (30). Thus, it can be hypothesized that deficient autophagy may also serve to augment ER stress and promote cell dysfunction, even cell death. In this study, when we used CQ, a lysosome inhibitor, to block the autophagic flux, this rendered cells susceptible to cell death, as evidenced by the increase in CHOP expression, which contributed to ER stress-induced apoptosis and decreased cell viability. Conversely, this effect was reversed by the activation of autopahgy using RAP. Therefore, our data clearly indicate that autophagy is induced in adipocytes subsequent to the activation of the ER stress-JNK pathway and plays a protective role against PA-induced cellular stress and death.

In addition to cell death, inflammation was also evident following treatment with PA. This was detected by measuring the expression levels of pro-inflammatory cytokine. To determine the role of autophagy in inflammation, we used the autophagy inhibitor, 3-MA. Notably, a further increase in the expression levels of MCP-1 and IL-6 was observed following pre-treatment with 3-MA compared to treatment with PA alone, which was also observed in response to treatment with the JNK inhibitor, SP600125. Our results strongly suggest that the activation of ER stress-JNK-autophagy plays a role in limiting PA-induced inflammation. The inhibition of autophagy in humans and mouse adipose tissue explants has been shown to lead to a significant increase in IL-1β, IL-8 mRNA expression and protein secretion (31). Previous studies about autophagy on inflammation is still unclear. Autophagy was demonstrated attenuate vascular endothelial inflammation through cAMP signaling pathway (32). The pro-inflammatory cytokine, IL-1β, is degraded in autophagosomes, which limits its availability for inflammasome-dependent activation (33). The suppression of autophagy may further increase inflammatory cytokine expression induced by ER stress, which interacts with a number of inflammatory signaling pathways (12). Further studies on the mechanisms of autophagy during inflammation are warranted.

In conclusion, in this study, we demonstrated that treatment with PA induced ER stress and, consequently, cell death and inflammation. The activation of autophagy occurred as an effective protective cellular response against PA-induced cellular stress in a JNK-dependent manner. Importantly, we present evidence of the role of autophagy in limiting PA-induced inflammation. This further validates the potential value of UPR-JNK targeted therapies as a promising approach for the treatment of obesity induced inflammation and insulin resistance.

## Abbreviations

ATF4: activating transcription factor 4
ATG: autophagy-related gene
AV: autophagic vacuole
CHOP: C/EBP homologous protein
CQ: chloroquine
ER: endoplasmic reticulum
IL-6: interleukin-6
IRE1: inositol-requiring enzyme 1
JNK: c-Jun N-terminal kinase
LC3: microtubule-associated protein 1 light chain 3
3-MA: 3-methyladenine
MCP-1: monocyte chemoattractant protein-1
mTOR: mammalian target of rapamycin
4-PBA: 4-phenyl butyrate
PERK: PKR-like endoplasmic reticulum kinase
eIF2α: eukaryotic translation initiation factor 2α
PA: palmitate
RAP: rapamycin
TLR: toll-like receptor
UPR: unfolded protein response
TNF-α: tumor necrosis factor-α
